# Risk factors associated with poor health outcomes for children under the age of 5 with moderate acute malnutrition in rural fagita lekoma district, Awi Zone, Amhara, Ethiopia, 2016

**DOI:** 10.1186/s40795-017-0208-5

**Published:** 2017-12-22

**Authors:** W/amilak Adamu, Dube Jara, Mulunesh Alemayehu, Sahai Burrowes

**Affiliations:** 1 SCI-MNCH, North Gondar, Ethiopia; 2 Department of Public Health, College of Medicine and Health Science Debre Markos University, P.O. Box 269, Debre Markos, Ethiopia; 3 Public Health Program, College of Education and Health Sciences, Touro University California, 1310 Club Drive, Mare Island Vallejo, CA 94592 USA

**Keywords:** Food security, Malnutrition, Treatment, Recovery

## Abstract

**Background:**

Left untreated, moderate acute malnutrition (MAM) in children can lead to severe acute malnutrition, stunting, developmental delays, and death. Despite recent progress the prevalence of malnutrition remains high throughout Ethiopia. The ability to make accurate prognoses and develop effective treatment strategies for children with MAM is currently limited and, as result, a significant proportion of children with MAM fail to recover even with treatment. We seek to address this limitation by assessing the risk factors for poor outcomes among children under the age of 5 with MAM in a rural area of Ethiopia’s Amhara Region. This region is considered relatively food secure and does not have food supplementation treatment programs.

**Methods:**

We conducted a prospective cohort study of 404 randomly sampled children, 0–59 months old stratified by household food security status. We followed the study children for approximately 2 months, assessing their health status; and used bivariate and multivariate Cox-proportional hazard regression models to identify risk factors for poor health outcomes.

**Results:**

Household food security was significantly associated with low recovery from MAM: 191 (60%) of children in food-insecure and 129 (40%) of children in food-secure households had poor health outcomes. The risk factors found to be significantly associated with poor health outcomes included the duration of exclusive breastfeeding (AHR 1.50, 95%CI: 1.05, 2.15), dietary diversity (AHR 1.74, 95%CI: 1.18, 2.54), and maternal mid-upper arm circumference (AHR=1.36, 95% CI: 1.04, 1.86). Children from pregnancies that were wanted but unplanned had 80% higher incidence of poor health outcomes than others, and children from pregnancies that were both unwanted and unplanned had more than double the incidence of poor health outcomes compared to their counterparts.

**Conclusion:**

We found that without treatment, the majority of children from food insecure households and over a third of children from food secure households did not recover from MAM. Maternal factors particularly the mother’s ability to plan her pregnancy were the main determinants of recovery in this study. Together these findings support arguments for targeting of nutrition support programs to vulnerable households regardless of regional food security status, and for closely integrating robust family planning, and antenatal care services with nutrition interventions.

## Background

Worldwide, approximately 5% of children under the age of five are affected by moderate acute malnutrition (MAM) [1]. Children with MAM are at increased risk of death and are more likely to suffer delays in their physical and cognitive development than children who have not experienced malnutrition [2, 3]. Recurrent episodes of MAM in children can also lead to later childhood stunting. In turn, stunting can affect the nutritional status of subsequent generations, resulting in “intergenerational growth failure”—a cycle of poor nutrition that perpetuates itself across generations with irreversible effect [4–6]. Moderate wasting is often described as the most important risk factor for childhood illness and mortality globally, and it is directly or indirectly responsible for more than half of all deaths in children under 5 year of age [7, 8].

Worldwide, approximately 165 million children under 5 are stunted—56 million in Africa. Both moderate and acute malnutrition remain a significant and persistent public health problem in Ethiopia [6]. According to the 2016 Demographic and Health Survey, 38% of children under 5 years of age were stunted; 10% had acute malnutrition; and 24% having moderate acute malnutrition. In the Amhara Region, in which this study was conducted, almost half (46%) of children are stunted [9].

Poor health outcomes due to MAM are not inevitable. With early adequate nutritional intervention and the prevention of infectious diseases, children with MAM can catch up their linear growth [5]. Such intervention is especially crucial in the child’s first 1000 days of life, a critical period for child growth and cognitive development [10]. Action to address MAM during this window of opportunity, particularly during the complementary feeding period (between 6 to 24 months), is crucial for preventing stunting and later cognitive and developmental delays [10, 11]. However, there is still debate about the proper strategies for addressing MAM at the national-level in a cost-effective manner.

A 2016 study [12] notes that the current Ethiopian strategy for managing MAM nationally is to restrict supplementary feeding programs (SFPs) for treatment to districts (*woredas*) that are chronically food-insecure. In areas not considered chronically food-insecure, there are no public SFPs; instead, providers rely on existing infant and child health interventions such as vitamin A supplementation, de-worming, water treatment, improved sanitation, and nutrition counseling. Researchers have noted that while this policy is logical in terms of efficiency, it may be failing to adequately address the problem of widespread malnutrition, because aggregate, average food security at the *woreda* level is may mask severe food insecurity at the household level [7, 12, 13]. Household food security has been shown to be associated with developing MAM and a failure to recover from MAM [14, 15]. Children with MAM need food of sufficient energy and nutrient density in order to recover from MAM and access to such food may be limited in food-insecure households [16]. There is, therefore, concern that the current policy may be inadequate to meet this pressing health threat.

Changing national strategies to prevent and treat MAM requires a solid understanding of the factors that are related to poor health outcomes for children with MAM, particularly children not receiving treatment. Such information facilitates the development of comprehensive programs containing complementary services and allows policy makers to better assess the tradeoffs involved in not providing SFPs for children with MAM. Unfortunately, the factors related to failure to recover from MAM, particularly for children not receiving treatment, are poorly understood. There is relatively strong evidence that household food insecurity, unhealthy environmental conditions, maternal under-nutrition and inadequate care and feeding practices contribute to the development of MAM [10]. Household wealth, maternal fertility, vitamin A supplementation, good hygiene practices, safe water source, having nutritional counseling, and the child’s Mid-Upper Arm Circumference (MUAC) at enrollment, have been found to be negatively associated with recovery in some studies [12, 17]. In others, the child’s age, gender, initial level of malnutrition and breastfeeding status were the important factors [18]. Household food insecurity and maternal workload have been found to be drivers of health outcomes in some studies but not in others [12]. This study wants to contribute to a better understanding of the risk factors for failure of children to recover from MAM in Ethiopia.

## Methods

### Study Setting

The study was conducted from February 2, 2016 to April 4, 2016 (the post-harvest, dry season) in the Fagita Lekoma *woreda*. Fagita Lekoma is one of 12 *woredas* in the Awi Zone, which is located in the Amhara Regional State. It is a rural *woreda* (20 of its 22 *kebeles* are rural) located 450 kms from Ethiopia’s capital, Addis Ababa [11]. We selected Fagita Lekoma because there are no SFPs in the *woreda*. The *woreda* has 6 health centers, only one of which provides outpatient care for severe acute malnutrition (SAM). The estimated population for the *woreda* is 156,671; with 36,435 households, and 21,213 children under the age of five. The five *kebeles* in this study had a total population of 22,682 [19].

### Study design and population

This community-based prospective cohort study was conducted among children with MAM aged 0–59 months. Children 59 months of age or younger with MAM that lived in the randomly sampled *kebeles* were eligible for recruitment. We excluded children older than 59 months; whose age was not known; without MAM; with no present mother or whose mother was unable to communicate with the study team; children who had health problems or disabilities that made it difficult to collect anthropometric measurements; and children with MAM who were receiving medical treatment.

### Sample size and sampling techniques

Because food security has been shown to be an important factor for predicting poor health outcomes in children with MAM we selected it as an “exposure” variable for sample size calculation and for the stratification of our Kaplan-Meier survival plots [12]. Households were categorized as food-secure and food-insecure based on Household Food Insecurity Access Scale (HFIAS) results that were from previous study [12]. We calculated our sample size using the double population proportion formula. Our assumptions were as follows: 37.78% children with MAM in food-secure households would have poor health outcomes [12] for an adjusted hazard ratio (AHR) of 1.39 for poor health outcome among food-secure compared to food insecure households [12]. We assumed a 95% two-sided confidence interval (CI), a statistical power of 80%, and a one-to-one allocation ratio of food-secure to food-insecure. Based on these assumptions, using EPI INFO 7 [20], we calculated a sample size 384. Allowing for an additional 5% non-response rate, the total sample size was 404 (202 for food-secure households and 202 for food-insecure households).

We randomly selected 5 *kebeles* from Fagita Lekoma’s 20 rural *kebeles* (25%) using a simple random sample lottery method. We then visited all households in the selected *kebeles* and screened all children aged 0–59 months (*n* = 2995) for their nutritional status. We used the conventional definition of MAM: having a weight-for-height (WFH) below the WHO median child growth standards (the child growth with Z-scores between -3SD to -2SD).

All children were assessed for WFH using WHO Anthro version 3.2.2 software and those with MAM were identified and registered. At this time we also categorized households as food insecure and food secure. We found 414 children with MAM (202 from food-secure and 212 from food insecure households). We retained all 202 children from food-secure households. We randomly selected 202 children from the remaining 212 food-insecure households using a lottery method. When there was more than one child with MAM in a household, we selected one of them using lottery method. The selected children were enrolled in the study and followed for two months.

### Study variables and measurement

Our outcome variable was whether, by the two-month follow up visit, a child had progressed to severe acute malnutrition (SAM); had not recovered from MAM, or had died. Children with any of these outcomes were categorized as having “*poor health outcomes*”.

We categorize children as having MAM, if at the second follow up visit, they had a weight-for-height/length (WFH/L) between -3 and - 2 Z-scores (-3SD to -2SD of the WHO median value), or WFH/L at 70–80% of the National Center for Health Statistics (NCHS), or had a MUAC measurement that was > = 11.5 cm <12.5 cm, without edema. Children whose MAM status did not change by the 2-month follow up period were categorized as not recovering. We categorized children as having SAM if, at the first or second follow-up visit, they had WFH/L below −3 SD of the WHO median value and/or (WFH/L) below 70% of the NCHS median value and/or MUAC <11.5 cm, with or without edema. Children were categorized as recovering if, at first and/or second follow up visit, they had WFH/L Z-scores > = -2SD of the WHO median value and/or WFH/L > =80% of the NCHS, and/or MUAC > = 12.5 cm) with no edema.

### Data collection methods

We collected data using a cross-sectional, structured, interviewer-administered questionnaire containing closed-ended questions and by taking anthropometric measurements of children and their mothers during home visits.

Our study began with the development of a project survey and the recruitment of project staff. Our survey was developed from standard, validated, English-language instruments that were translated to into Amharic. We recruited 2 health officers to supervise data collection, and 10 health extension workers and 3 nurses to act as data collectors. All spoke Amharic, the local language. We then conducted one-day training on how to collect the data for the data collectors and supervisors and then pre-tested the questionnaire in a *kebele* that was adjacent to our study *kebeles*, with 20 households (5% of our sample size).

The study had three data collection points: we collected baseline survey and anthropometric data during community-based nutritional screening for all children 0–59 months of age in our 5 sampled *kebeles*. We used the HFIAS to measure food security for stratifying the sample [21]. This tool is the current standard for assessing household-level food security and has been validated for use in Ethiopia [22]. Households that were enrolled in the study were visited once monthly for 2 months, during which mothers were asked follow up survey questions and anthropometric measurements of the study children were taken.

The survey contained questions on socio-economic factors, demographic risk factors, child characteristics, child-care practices, maternal characteristics, and environmental risk factors. We recorded the child’s vaccination status by reviewing immunization cards when these were available, or by using the mother’s recall. We checked bacille Calmette-Guerin vaccination by observing whether there was scar on the child’s arm.

We used procedures stipulated by the WHO to take anthropometric measurements [23]. Before measuring children we established their age, using a local event to establish the child’s birth period. Mothers were asked whether the child was born before or after certain major events until a fairly accurate age was pinpointed. If we were not able to determine the child’s age accurately, the next child in the household was recruited. We measured body length of children age up to 23 months (or those who were older but too ill to stand) in the recumbent position, without shoes, reading the length to the nearest 0.1 cm or 1 mm using a horizontal wooden length measuring board/sliding board. We measured MUAC for both the study children and their mothers. MUAC was measured on left mid upper arm half way between the olecranon process and acromion process using a non-stretchable strap, to the nearest 1 mm.

### Quality control measures

We checked the calibration of the measurement scale by weighing a 2-kg stone after each child measurement and after moving the scale from one household to another. Then the scale indicators were checked against a zero reading before and after weighing every child and mother. Only one observer was used for each subject. Mothers and children were required to wear only light clothing in order not to skew the weight results. The project principal investigator reviewed collected data on a daily basis, and returned records with possible errors to the data collectors for correction.

### Data processing and analysis

The collected data were checked for completeness, consistency and entered using EPI-data software; then the data were exported to SPSS version 20 for analysis [24]. Descriptive analysis such as Kaplan-Meier survival curves and log-rank test statistics were used to describe important variables of the study and compare the outcome variables. A Cox-regression model was fitted to identify risk factors for poor health outcomes of MAM. All predictors that were associated with the outcome variable in bivariate analysis at *p*-values of 0.20 or lower were included in our multivariate Cox-regression models. Crude and adjusted hazard risks with their corresponding 95% confidence intervals were computed. Variables with p-values <0.05 were considered statistically significant risk factors in this study.

## Results

A total of 202 children with MAM in food-secure and 202 in food-insecure households were enrolled in the study with no loss to follow-up, for an overall response rate of 100%.

### Socio-demographic and economic characteristics

The sample was homogenous in terms of religion and ethnicity (100% Awi ethnicity and Ethiopian Orthodox). Most households were male-headed: 182 (90%) in-food secure, and 180 (89.1%) in food-insecure households. Approximately two-thirds of households had 5 members or more: 130 (64.35%) in food secure, and 138 (68.31%) in food-insecure households. Approximately 40% of sample fathers and 64% of mothers were illiterate. Slightly less than half (46%) of mothers report that their husbands were in charge of decision-making regarding the use of household money. Almost all households earned less than 750 Ethiopian birr per month (approximately 32 US dollars) (see Table 1).

**Table 1:** Socio-demographic and economic household characteristics in Fagita District, Awi Zone, Amhara, Ethiopia, 2016

Variables	Description	Moderate Acute Malnutrition			
		Food-Secure Households		Food-Insecure Households	
Sex HH head	Female	20	9.90%	22	10.89%
	Male	182	90.0%	180	89.1%
Age of male HH head	15-39	27	13.36%	27	13.36%
	30-39	72	35.64%	64	31.68%
	40-49	75	37.12%	82	40.59%
	50andabove	8	3.90%	7	3.46%
Marital Status of HH Head	Married	182	90.09%	180	89.1%
	Divorced	12	5.94%	16	7.92%
	Widowed	8	3.96%	6	2.97%
No. of person in household	<5	72	35.64%	64	31.68%
	5 and above	130	64.35%	138	68.31%
Father’s Educational Status	Can't Read &write	91	45.04%	80	39.6%
	Can Read &Write and above	91	45.04%	100	49.5%
Maternal Educational Status	Can't read &write	137	67.8%	121	59.9%
	Can Read &Write and above	65	32.17%	81	40.09%
Maternal Occupational Status	Farmer	200	99%	201	99.5%
Who Decides Money Use	Mainly spouse	20	9.9%	24	11.880%
	Mainly husband	94	46.53%	98	48.53%
	Both	66	32.67%	56	27.72%
	Only husbanded	22	10.89%	24	11.88%
Monthly Income	<750	192	95.05%	195	96.50%
	750+	10	4.95%	7	3.50%

### Child characteristics

Approximately half of the study children were female. The vast majority had been full term births (81.69%), that occurring within a 24–48 month birth interval (52.72%). The plurality (47.28%) were between the age of 12–23 (See Table 2).

**Table 2:** Child characteristics in Rural Fagita District, Awi Zone, Amhara, Ethiopia, 2016

Variables	Description	Moderate acute malnutrition			
		Food-Insecure Households		Food-Insecure Households	
Child’s sex	Female	101	50.0%	111	54.95%
	Male	101	50.0%	91	45.04%
Child’s age	0-11	29	14.35%	31	15.34%
	12-23	99	49.00%	102	50.45%
	24-35	50	24.75%	44	21.78%
	36-47	18	8.90%	18	8.90%
	48-59	6	2.90%	7	3.40%
Birth interval	0-23 months	95	47.02%	85	42.07%
	24+ months	107	52.97%	117	57.92%
Gestational age	<9month	28	13.86%	46	22.77%
	9+ month	174	86.13%	156	77.22%
Illness in the last 2 weeks	149	73.76%	147	72.77%	
	53	26.23%	55	27.22%	
Frequency of health problems	Fever	9	16.98%	5	9.00%
	Cough	6	11.32%	5	9.00%
	Fever & cough	13	24.52%	25	45.45%
	Vomiting &diarrhea	25	47.16%	20	36.36%

### Child care characteristics

We found high rates of exclusive breastfeeding in children from food-insecure households (83.47%) but more modest rates in among households with higher food security (57.14%). Roughly 80% had been given breast milk immediately after birth. More than half of the children (55.44% and 59.9% in food-secure and insecure households respectively) were being breastfed at the time of data collection.

A larger proportion of children in food-secure households had not been given pre-lactation feeding than in food-secure households (86.16% vs. 82.67%). The most common pre-lactation foods were water, butter, and milk. At the time of the survey, 88.09% of mothers in the food-secure households and 73.59% from food-insecure households reported having eaten from less than 4 food groups within the last 24 h (see Table 3).

**Table 3:** Child caring characteristics in Rural Fagita District, Awi Zone, Amhara, Ethiopia, 2016

Variables	Description	Moderate Acute Malnutrition			
		Food-Secure Households		Food-Insecure Households	
Breast fed	No	90	44.55%	81	40.09%
	Yes	112	55.44%	121	59.9%
Time to initiate breast feeding	Immediately	92	82.14%	97	80.16%
	>=1Hr of Delivery	20	17.85%	24	19.83%
Frequency of breast feed in 24hrs	<8 time/day	21	18.75%	23	19.00%
	8-12 time/day & above	91	81.25%	98	80.99%
Exclusively breast feed	< 6 month	48	42.85%	20	16.52%
	>=6 month	64	57.14	101	83.47%
Pre-lactation food/fluid	No	174	86.13%	167	82.67%
	Yes	28	13.86%	35	17.32%
Material used to feed the child	Cup	27	96.42%	30	85.74%
	Bottle	1	3.57%	5	14.28%
Who is in charge of the baby’s Feeding	Mother	199	98.51%	198	98.01%
	Sister	3	1.48%	3	1.48%
Sickness in the last 2weeks	No	191	94.55%	190	94.05%
	Yes	11	5.44%	12	5.94%
Breast feeding until 1 year(6-23 month)	No	1	0.78%	1	0.78%
	Yes	126	99.2%	127	99.21%
Time to start complementary feeding(6-23month)	1-5months	24	19.04%	29	22.83%
	6months	89	68.99%	84	66.14%
	7-12months	14	11.11%	14	11.02%
Took soft thick (semi solid) porridge (6-8 months)	No	42	10.4%	51	12.6%
	Yes	8	2.0%	9	2.2%
Frequency the child take semi-solid porridge(6-8 month)	1-2 time/day	3	37.5%	4	44.44%
	2-3 time/day	4	50.00%	4	44.44%
	3-4 time/day	1	12.5%	1	11.11%
Took soft thick (semi solid) porridge (9-11 months)	No	41	75.92%	52	81.25%
	Yes	13	24.07%	12	18.75%
Family food consumption	No	39	34.51%	39	33.33%
	Yes	74	65.48%	78	66.66%
Frequency of family food	1-2 time/day	8	10.81%	4	51.28%
	2-3 time/day	64	86.48%	71	91%
	3-4 time/day	2	2.70%	3	3.84%
The number of MDD intake	Legumes & Nut	4	3.10%	6	4.68%
	Diary Products	79	62.20%	86	67.18%
	Grain, rut, tuber, diary product, Legume & Nuts	38	29.92%	26	20.31%
	<4 food groups	111	88.09%	101	79.52%
	4+ food groups	15	11.91%	31	20.48%
MDD in 24 hours stop breast milk	No	113	88.97%	118	92.18%
	Yes	14	11.02%	10	7.81%
Immunized	No	0	0%	1	0.79%
	Yes	127	100%	127	99.21%
Vitamin A supplementation	No	26	20.47%	26	20.31%
	Yes	101	79.52%	102	79.69%
Vaccines received	Only Penta1 and 2 K	3	2.3%	4	3.12%
	Fully	107	84.25%	110	85.93%
	BCG, penta1-penta3	12	11.81%	6	4.68%
	Not Sure	5	3.90%	4	3.12%

### Maternal characteristics

Many mother in the study were either malnourished or highly vulnerable to malnutrition. The majority of mothers were 30–39 years old. A significant proportion had a chronic energy deficiency (30% in food-secure households and 43% in food-insecure households). According to the MUAC measurements, almost half of the mothers (49%) from food-secure households and more than two-thirds (70%) of mothers from food insecure household were acutely malnourished. Almost 70% of mothers had experienced 2 to 5 pregnancies. Most had not been able to eat extra meals during these pregnancies or during lactation. We found large differences between food-secure and insecure mothers in whether the child enrolled in the study had come from a planned and/or wanted pregnancy. While a large majority (73%) of pregnancies in food-secure households had been wanted and planned, only 56% of pregnancies in food-insecure households were wanted and planned. This difference was statistically significant (see Table 4).

**Table 4:** Maternal characteristics in Rural Fagita District, Awi Zone, Amhara, Ethiopia, 2016

Variables	Description	Moderate Acute Malnutrition			
		Food-Secure Households		Food-Insecure Households	
Mother’s age	15-29	50	24.75%	45	22.27%
	30-39	120	59.40%	120	59.40%
	40-49	32	15.84%	37	18.31%
BMIin kg/m2	<18.5kg/m2(CED)	60	29.70%	87	43.06%
	18.5+kg/m2(NM)	142	70.29%	115	56.93%
MUAC in centimeter	<23cm	99	49.00%	141	69.8%
	>=23cm	103	51.00%	61	30.2%
Gravidity	1	28	13.86%	25	12.37%
	2-5	136	67.32%	138	68.31%
	>5	38	18.81%	39	19.30%
Parity	1	28	13.86%	25	12.37%
	2-5	137	67.82%	139	68.81%
	>5	37	18.31%	38	18.81%
Extra meal during pregnancy	No	129	63.86%	138	68.31%
	Yes	73	36.13%	64	31.68%
Number of extra meals during pregnancy	<2 time/day	44	60.27%	41	64.06%
	>= 2 time/day	29	39.72%	23	35.93%
Extra meals during lactation	No	137	67.82%	145	71.78%
	Yes	65	32.17%	57	28.21%
Number of extra meals during pregnancy	<2 time/day	45	69.23%	35	61.4%
	>= 2 time/day	20	30.76%	22	38.59%
Pregnancy type	Wanted & planned	148	73.26%	113	55.94%
	Wanted & unplanned	33	16.33%	61	30.19%
	Unwanted & unplanned	21	10.39%	28	13.86%

### Health Outcomes

**Fig. 1:**
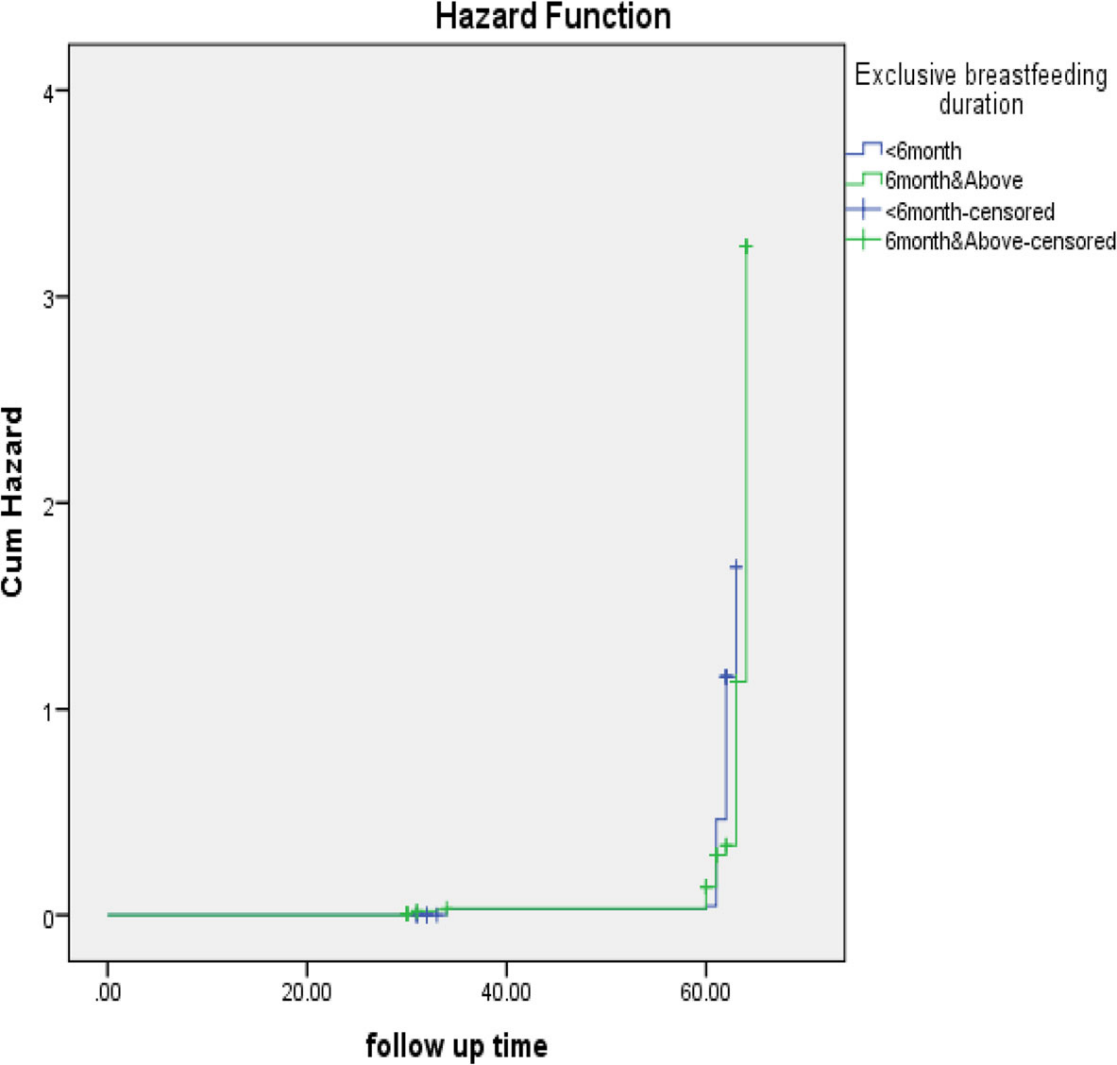
Kaplan-meier hazard estimation of moderate acute malnutrition children by duration of exclusive breastfeeding, in Fagita District, Awi Zone, Amhara, Ethiopia, 2016

Children enrolled in the study were followed for 64 days. The minimum and maximum follow up days were 31 and 64 days, respectively. By the last follow up visit, 320 (79%) had poor health outcomes: 191(60%) from food-insecure and 129 (40%) from food-secure households. As follow up time increased, the hazard of the children developing poor health outcomes was higher for those who had been exclusively breastfed for less than 6 months comparing with those who had not (please see Fig. 1).

### Median recovery times

The median recovery time was 62 days for children from food secure households (95% CI 61.65–62.35) and 63 days for children from food insecure households (95% CI 62.78–63.22). The overall median recovery time was 63 days (95%CI 62.83–63.17) (see Table 5).

**Table 5:** Median recovery time for children with moderate acute malnutrition in Rural Fagita District, Awi Zone, Amhara, Ethiopia, 2016

Food security Status	Mean	Std. Error	95% CI	Median	Std. Error	95% CI
Food secure	62.29	0.097	(62.10, 62.48)	62.0	0.178	(61.65, 62.35)
Food insecure	61.67	0.432	(60.82, 62.51)	63.0	0.111	(62.78, 63.22)
Overall	61.98	0.225	(61.54, 62.42)	63.0	0.087	(62.83, 63.17)

Recovery time was one day longer for children whose mothers had low baseline MUAC measurements (63 days for mothers with MUAC <23 cm (95% CI 62.83, 63.17) versus 62 days for mothers with MUAC > = 23 cm (95% CI 61.62, 62.38). Children with well-nourished mothers from food-secure households had the fastest recovery. Their median recovery time was 61 days (95% CI 60.65, 61.35) compared to 63 days (95% CI 62.44, 63.56) for children whose mothers were well-nourished from food-insecure households (see Table 6).

**Table 6:** Median recovery time for children with moderate acute malnutrition by Maternal MUAC in Rural Fagita District, Awi Zone, Amhara, Ethiopia, 2016

MUAC in centimeter	Food security Status	Mean	Std. Error	95% CI	Median	Std. Error	95% CI
<23cm	Food secure	62.78	0.114	(62.56, 63.00)	63.0	0.130	(62.75, 63.26)
	Food insecure	61.24	0.661	(59.95, 62.54)	63.0	0.119	(62.77, 63.23)
	Overall	61.90	0.384	(61.15, 62.65)	63.0	0.088	(62.83, 63.17)
>= 23cm	Food secure	61.78	0.141	(61.50, 62.06)	61.0	0.181	(60.65, 61.35)
	Food insecure	62.45	0.167	(62.12, 62.77)	63.0	0.285	(62.44, 63.56)
	Overall	62.08	0.111	(61.86, 62.30)	62.0	0.194	(61.62, 62.38)

### Risk factors for poor health outcomes due to MAM

In order to identify variables for our multivariate regression models we run bivariate Cox regressions containing variables that had been shown to be risk factors for poor health outcomes in past studies. We retained all variables that were found to be statistically significant at the 20% level or lower. We then run a multivariate Cox regression model containing all of these variables and retained the variables that were associated with poor health outcomes in children at the 5% level of significance. These four variables were: whether the mother had breastfed the child exclusively, the diversity of the child’s diet, maternal malnutrition, and whether the mother’s pregnancy had been planned and/or wanted (see Table 7).

**Table 7:** Risk factors for poor health outcomes of moderate acute malnutrition in multivariate cox regression in Rural Fagita District, Awi Zone, Amhara, Ethiopia, 2016

Variable	Moderate Acute Malnutrition		CHR at 95% CI	AHR at 95% CI	*p*-Value
	Food-Secure Households	Food-Insecure Households			
Exclusive breast feeding					
<6 month	48 (42.85%)	20 (16.52%)	1.89 (1.36,2.62)	1.50 (1.05, 2.15)	0.027
>=6 month	64 (57.14%)	101 (83.47)	1	1	
Dietary diversity					
<4 food groups	111 (88.09%)	101 (75.59%)	1.67 (1.19,2.37)	1.74 (1.184,2.54)	0.005
>=4 food groups	15 (11.90%)	31 (24.40%)	1	1	
Maternal MUAC					
<23cm	99(49.00%)	141 (69.8%)	1.45 (1.14, 1.82)	1.36 (1.05,1.86)	0.044
>=23cm	103 (51%)	61(30.19%)	1	1	
Pregnancy type					
Wanted & planned	148 (73.26%)	11 3(55.94%)	1	1	
Wanted & unplanned	33 (16.33%)	61 (30.19%)	1.49 (1.16,1.92)	1.80 (1.27,2.26)	0.001
Unwanted & unplanned	21 (10.39%)	28 (13.86%)	1.66 (1.21,2.28)	2.19 (1.44,3.31)	0.001

The risk of having poor health outcomes among children who had been exclusively breastfed for less than 6 months were 50% times higher than among children who had exclusively breastfed for 6 months or more (AHR = 1.50, 95% CI: 1.05, 2.15). Children with MAM had 74% higher risk of having poor health outcomes if they had eaten less than 4 food groups in the last 24 h compared with children whose diet was more diverse (AHR = 1.74, 95% CI: 1.184, 2.54). Maternal nutritional status was significantly associated with poor health outcomes. Children whose mothers had MUAC measurements less than 23 cm had 36% higher risk of having poor health outcomes than children whose mothers had measurement that were > = 23 cm (AHR = 1.36, 95% CI: 1.04,1.86). We also found a surprisingly strong association between whether a pregnancy was planned and/or wanted and the risk of poor health outcomes. Poor health outcomes were 80% higher among children born of unplanned pregnancies that were wanted compared children from wanted and planned pregnancies (AHR = 1.80, 95% CI: 1.27, 2.26). And the risk of poor health outcomes was 119% higher among children born of pregnancies that were both unwanted and unplanned (AHR = 2.19, 95% CI: 1.44, 3.31).

## Discussion

This study set out to explore the risk factors associated with poor health outcomes in children with MAM in a food-secure region of Ethiopia with little access to supplemental feeding programs. We found that without treatment, the vast majority of children (79%) did not recover from MAM and that the proportion of children who failed to recover was significant even in food-secure households (approximately 40%). This high level of non-recovery is concerning particularly because most of the children in our study were in the critical, first 1000 days of life. Studies have found that children in this vulnerable age range (0–23 months) are relatively more prone to moderate wasting, which is often exacerbated by bouts of diarrhea and respiratory illness, deteriorating into more severe illness [10].

Two of the risk factors found in our study were consistent with those found previously, namely, maternal nutritional status and the diversity of the child’s diet [12]. However, several of our findings were unusual. Many of the factors found to be associated with the development of, or the recovery from, moderate acute malnutrition in other studies were not found to be significant factors in risk of non-recovery in our study. These include the child’s age, household wealth [25], maternal education [11], household income [11], drinking water source, sanitation (e.g., hand washing practices, appropriate waste disposal and latrine availability), household size [12], having received vitamin A supplementation or de-worming interventions [26] . This discrepancy may be due to the fact that our study took place during the post-harvest season when relative abundance of food might have mitigated or washed out the impact of other factors. The positive impact of high rates of exclusive breastfeeding that we observed in this study may have also played a similar role.

However, the rates of poor health outcomes for children with MAM observed in this study were quite high, and indeed much higher than that observed in previous Ethiopian research. For example, a similar study, recently conducted in Jimma found that roughly 62% of children with MAM either deteriorated or remained with MAM compared to our 79% rate [12]. Their lower rates may be due to differences between our sample in the age ranges of children (our sample was considerably younger than these previous studies) and the fact that the follow up period in the Jimma study was longer than ours, which gave children a more time to recover. There may have also been differences in our samples the duration of exclusive breastfeeding and the proportion of pregnancies that were wanted and planned. .

Indeed, the strength of association between poor health outcomes and whether a pregnancy was planned and/or wanted was one of the surprising findings of this study. While associations between fertility and overall rates of childhood malnutrition have been observed in numerous studies [27, 28] there has been less evidence of a relationship between the ability to plan pregnancy and women’s ability to effectively care for children with MAM.

## Conclusions and Recommendations

This study finds that, without intervention, the majority of children with MAM fail to recover. After a 2-month period, most either remain moderately malnourished or their condition deteriorates. Those from food-insecure households were more likely to have poor health outcomes than those from food secure households. These poor health outcomes are negatively associated with the duration of exclusive breastfeeding, minimum dietary diversity, maternal MUAC and whether pregnancies were planned and/or wanted. Based on the finding of the study the following recommendations were forwarded.

The high level of non-recovery found in our study suggests the need for ongoing, intensive, community-based nutrition education programs and nutritional surveillance to tackle the problem. It also suggests that children with MAM should be managed by targeted supplemental feeding programs, regardless of the food security status of the *woreda*. This would require the additional investment of scare resources. It could also be an important preventative measure that reduces the future outlay of resources for more expensive interventions to treat severe acute malnutrition and its co-morbidities. Our findings also suggest that it will be crucial to link these additional nutritional interventions for children with programs that provide intensive, high quality, prenatal and antenatal care for women. Our findings suggest that these programs should provide maternal nutritional support, counseling support for exclusive breastfeeding and diversifying childhood diets, and, most importantly, family planning counseling and provision of contraception, (particularly long-acting contraception), and safe abortion services when needed. This, in turn will require closer collaboration between stakeholders in reproductive health services, and those working in infant and young child feeding programs.

In order to inform the development of these programs, further study on the management of MAM should be conducted to explore risk factors not measured in the present study and to in particular, to address two of the main limitations on this study, its limited geographic scope, and its short time frame which did not include the pre-harvest and harvest seasons.

## Abbreviations

AHR: Adjusted Hazard Rate
BMI: Body Mass Index
CI: Confidence interval
CMAM: Comprehensive Moderate Acute Malnutrition
EBF: Exclusive breastfeeding
HEWs: Health Extension Workers
HFIAS: Household Food Insecurity Access Scale
MAM: Moderate Acute Malnutrition
MDD: Minimum Dietary Diversity
MUAC: Mid Upper-arm Circumference
NCHS: National Center for Health Statistics
SAM: Severe acute malnutrition
SD: Standard Deviation
SPSS: Statistical Package for Social Sciences
WFH: Weight for height
WFL: Weight for length
WHO: World Health Organization
